# A meta-analysis of Boolean network models reveals design principles of gene regulatory networks

**DOI:** 10.1126/sciadv.adj0822

**Published:** 2024-01-12

**Authors:** Claus Kadelka, Taras-Michael Butrie, Evan Hilton, Jack Kinseth, Addison Schmidt, Haris Serdarevic

**Affiliations:** ^1^ Department of Mathematics, Iowa State University, Ames, IA 50011, USA.; ^2^ Department of Aerospace Engineering, Iowa State University, Ames, IA 50011, USA.; ^3^ Department of Computer Science, Iowa State University, Ames, IA 50011, USA.; ^4^ Bioinformatics and Computational Biology Program, Iowa State University, Ames, IA 50011, USA.

## Abstract

Gene regulatory networks (GRNs) play a central role in cellular decision-making. Understanding their structure and how it impacts their dynamics constitutes thus a fundamental biological question. GRNs are frequently modeled as Boolean networks, which are intuitive, simple to describe, and can yield qualitative results even when data are sparse. We assembled the largest repository of expert-curated Boolean GRN models. A meta-analysis of this diverse set of models reveals several design principles. GRNs exhibit more canalization, redundancy, and stable dynamics than expected. Moreover, they are enriched for certain recurring network motifs. This raises the important question why evolution favors these design mechanisms.

## Teaser

Leveraging the collective knowledge about gene regulatory networks yields remarkable insights into their structure and function.

## INTRODUCTION

Gene regulatory networks (GRNs) describe how a collection of genes governs key processes within a cell. Understanding how GRNs perform particular functions and do so consistently despite ubiquitous perturbations constitutes a fundamental biological question (*1*). Over the past two decades, a variety of design principles of GRNs have been proposed and studied, with a focus on discovering causal links between network form and function.

GRNs have been shown to be enriched for certain subgraphs with a specific structure, so-called network motifs, like feed-forward loops (FFLs), feedback loops (FBLs) but also larger subcircuits (*2*–*5*). Theoretical studies of the dynamic properties of these motifs revealed specific functionalities (*6*, *7*). For example, coherent FFLs can delay the activation or inhibition of a target gene, while incoherent ones can act as accelerators (*8*). Other hypothesized design principles include redundancy in the regulatory logic (*9*, *10*) and a high prevalence of canalization (*11*, *12*). Canalization, a concept originating from the study of embryonal development (*13*), refers to the ability of a GRN to maintain a stable phenotype despite ample genotypic as well as environmental variation.

Over the past decades, Boolean networks [reviewed in (*14*)] have become an increasingly popular modeling framework for the study of biological systems, as they are intuitive and simple to describe. When data are sparse, as is still often the case for less-studied organisms and processes, complicated models (e.g., continuous differential equation models), which harbor the potential for quantitative predictions, cannot be appropriately fitted to the data because of their high number of parameters (*15*). In this case, Boolean network models can often still yield qualitative results.

Static network models are composed of (i) a set of considered nodes (genes, external parameters, etc.) and (ii) a wiring diagram (also known as dependency graph), which describes which node regulates which and often also contains information about the respective type of regulation (positive because of e.g. transcriptional activation versus negative because of e.g., inhibition). A dynamic Boolean network model has these same features but obtains its dynamics from an additional set of update rules (i.e., Boolean functions) that describe the regulatory logic governing the expression of each gene. Each gene is either on (i.e., high concentration, expressed) or off (i.e., low concentration, unexpressed) and time is discretized as well.

Large, genome-wide static transcriptional network models can be easily assembled from existing databases like TRANSFAC (*16*), JASPAR (*17*), or RegulonDB (*18*), by simply considering all known transcriptional regulations for a given species. However, information about the network topology alone provides only an incomplete understanding of a system, which is intrinsically dynamic. The formulation of dynamic models such as Boolean networks requires a careful calibration of the update rules by a subject expert. Therefore, all dynamic Boolean GRN models published thus far focus on specific biological processes of interest and contain only those genes involved in these processes (*19*). Moreover, most dynamic models have been published over the course of the past 12 years, as biological data needed for an accurate model description has become increasingly available. Over the course of the past few years, researchers have started to leverage the collection of these models to gain insights into specific aspects of GRNs such as the role of nonlinearity (*20*), canalization (*21*, *22*), or the connection between canalization and criticality (*12*, *23*, *24*).

Here, we describe a comprehensive meta-analysis of the largest repository of published, expert-curated Boolean GRN models assembled thus far. This provides a detailed understanding of the design principles of GRNs that are potentially conserved across organisms and can help explain how GRNs operate smoothly and perform particular functions.

## RESULTS

Using the biomedical literature search engine Pubmed, we created a database of 163 Boolean GRN models. To avoid introducing bias into the meta-analysis, we only included expert-curated models where the nodes and the update rules were selected by hand and not by a prediction algorithm or where default choices like threshold rules were used throughout. We further included only one version of highly similar models. This led to the exclusion of 41 models (see Materials and Methods for details), resulting in a total of 122 models used in the meta-analysis, of which 61 are included in the Cell Collective (*19*) and 61 are not. The models describe the regulatory logic underlying a variety of processes in numerous species across multiple kingdoms of life (animals: 93, plants: 10, fungi: 9, bacteria: 9; data S1).

**Fig. 1.**
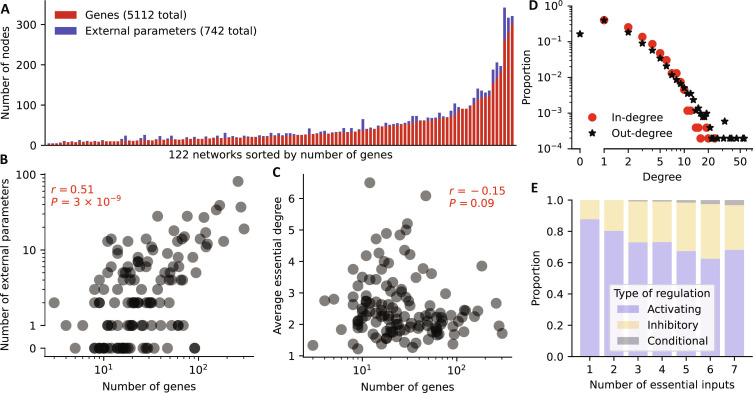
Summary statistics of the analyzed GRN models. (**A**) Plot of the number of genes and external parameters for each model sorted by number of genes. (**B** and **C**) For each model, the number of genes is plotted against (B) the number of external parameters and (C) the average essential in-degree of the genes. The Spearman correlation coefficient and associated *P* value are shown in red. (**D**) In-degree (red circles) and out-degree (black stars) distribution derived from all 5112 update rules. (**E**) Prevalence of each type of regulation (activation, blue; inhibition, orange; conditional, gray) stratified by the number of regulators (*x* axis). Nonessential regulations are excluded.

The models contain different types of nodes. Some nodes are unregulated (i.e., they do not receive incoming edges in the wiring diagram) and remain thus constant over time. We refer to these nodes as external parameters because they frequently represent abstract external conditions such as the temperature or pH level. Most other nodes represent genes. We therefore refer to all nodes that receive incoming edges in the wiring diagram as genes but acknowledge that this is a simplification as some regulated nodes also represent molecules or abstract phenotypes such as cell proliferation or apoptosis. The 122 investigated GRN models ranged in size from 3 to 302 genes (mean = 41.9, median = 23), and encompassed a total of 5112 genes as well as 742 external parameters (Fig. 1A). Some genes (as well as external parameters) appeared in multiple models (data S2), with *AKT* appearing the most frequently, in 33 models.

A majority of the investigated models (94, 77%) contained external parameters. As expected, network models with more genes contained on average more external parameters (ρ_Spearman_ = 0.51, Fig. 1B). On the other hand, the size of a network was slightly negatively correlated with the average connectivity, i.e., the average number of regulators per gene (ρ_Spearman_ = −0.15, Fig. 1C). The average connectivity differed widely across the 122 models; we observed a range of [1.22,6.5] and a mean average connectivity of 2.56 (median = 2.27). The degree distribution of a random graph, in which the edges are distributed randomly, is a Poisson distribution (*25*). When considering all update rules separately, we identified that the in-degree distribution resembled a Poisson distribution, while the out-degree distribution had a power-law tail (Fig. 1D), as has been observed for many diverse types of networks (*25*, *26*), including the yeast transcriptional regulatory network (*27*). The tails of the two degree distributions differed substantially; we found many more instances of high out-degree versus high in-degree, highlighting the presence of key transcription factors that act as network hubs (*28*, *29*).

Next, we investigated the prevalence of different types of regulations. If gene A regulates gene B, there are three possibilities: (i) Gene A may activate gene B, meaning that an increased expression of gene A (i.e., a change from 0 to 1 in the Boolean world) leads to an increased expression of gene B for some states of the other regulators, and possibly no change in B for other states of the other regulators. (ii) Gene A may inhibit gene B, meaning that an increase in A leads to a decrease in B for some states of the other regulators, and possibly no change in B for other states of the other regulators, and (iii) gene A’s effect on gene B may be conditional (i.e., not monotonic), meaning that for some states of the other regulators, A activates B, while for other states of the other regulators, A inhibits B. Except for two rules with more than 20 inputs, we investigated all update rules, resulting in a total of 12,514 analyzed regulators (where some genes regulate more than one gene and each such regulation is considered separately). The majority of regulations were activations (9237, 73.8%), followed by inhibitions (2951, 23.6%) and conditional behavior (111, 0.9%). Regulatory networks in eukaryotes operate mainly by activation of otherwise inactive promoters (*30*). On the contrary, many promoters in prokaryotes are by default expressed and require repressors to reduce gene activity (*31*). Most of the considered GRN models are eukaryotic (data S1), which could serve as an explanation of the increased prevalence of activation. Unexpectedly, we found, however, the largest proportion of activating interactions in bacterial (i.e., prokaryotic) GRN models (656/785 = 83.6%), compared to 7844/10,367 = 75.7%, 324/482 = 67.2%, 401/640 = 62.7% in GRNs of animals, fungi, and plants, respectively. Activation further seemed particularly prevalent in situations where a gene’s state is determined by one or only a few regulators (Fig. 1E), irrespective of the considered kingdom (fig. S1).

We found that 215 of the 12,514 regulators (1.7%) contained in the ensemble of Boolean update rules were nonessential. That is, these regulators appeared in the published rules but did not have any effect on the output. For example, the Boolean update rule (*X* AND *Y*) OR *X* simplifies to *X*; *Y* is therefore a nonessential regulator. The nonessential regulators were spread across 23 (18.9%) models and 120 (2.3%) update rules, i.e., some update rules contained more than one nonessential regulator. In one extreme case, an update rule with 12 different inputs simplifies to the Boolean zero function. Figure S2 shows the discrepancy between the number of inputs in the published update rules and the number of inputs that have an actual effect on the dynamics. In the rest of this paper, only essential regulators were considered.

### Canalization

The concept of canalization, already introduced in the 1940s in the context of embryonal development (*13*), has been proposed as a possible explanation for the remarkable stability of GRNs in the face of ubiquitous perturbations (*32*, *33*). Accordingly, Boolean canalizing functions have been proposed as suitable update functions in Boolean GRN models (*34*). Recently, the class of canalizing functions has been further stratified and studied (*35*, *36*). Some smaller studies support the general hypothesis by revealing an overabundance of canalizing functions in GRN models (*12*, *37*), but a rigorous, comprehensive analysis that considers various types of canalization is still missing.

A canalizing function has at least one input variable such that, if this variable takes on a certain “canalizing” value, then the output value is already determined, regardless of the values of the remaining input variables. If this variable takes on another value, and there is a second variable with this same property, the function is two-canalizing. If *k* variables follow this pattern, the function is *k*-canalizing (*35*), and the number of variables that follow this pattern is the canalizing depth of the function (*38*). If the canalizing depth equals the number of inputs (i.e., if all variables follow the described pattern), the function is also called a nested canalizing function (NCF).

**Fig. 2.**
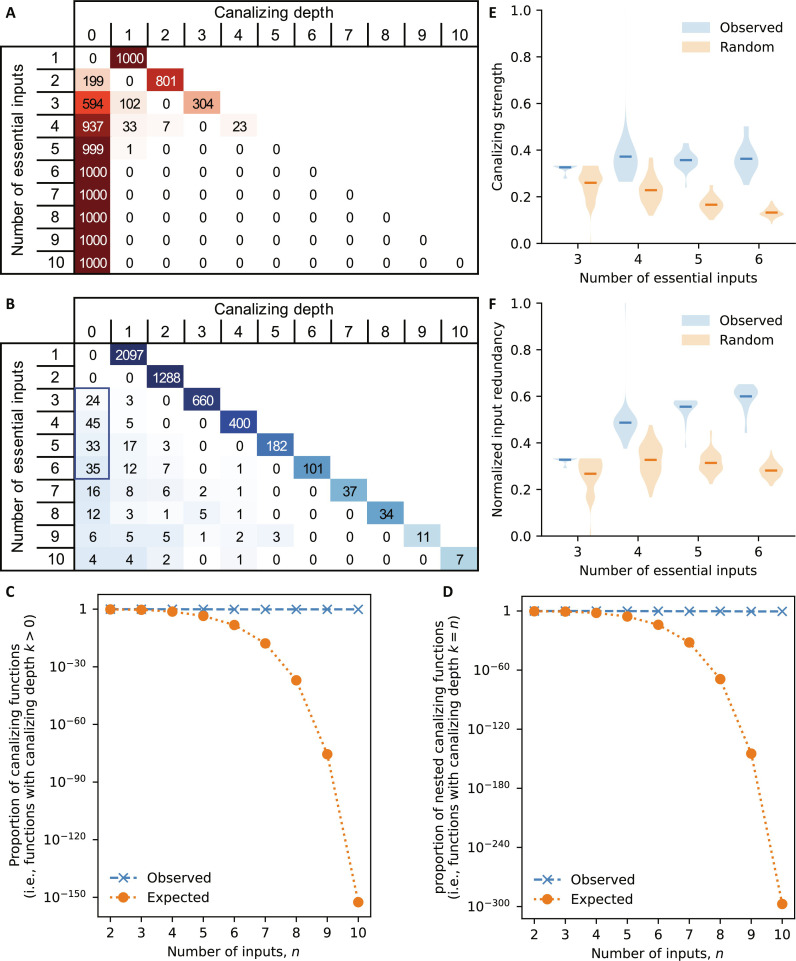
High prevalence of canalization. (**A**) Expected distribution of the canalizing depth for random Boolean functions for different numbers of essential inputs (1–10), based on 1000 random functions each. (**B**) Stratification of all identified update rules based on the number of essential inputs (rows) and the canalizing depth (columns). Update rules with more than 10 inputs were omitted here; table S1 contains the full analysis. The color gradient in (A) and (B) is computed separately for each row. (**C** and **D**) For Boolean functions with 2 to 10 (not necessarily essential) inputs, the proportion of (C) canalizing functions and (D) NCFs observed in published expert-curated GRN models (blue x) is compared to the expected proportion (orange dots), which is computed using explicit formulas for the number of canalizing and nested canalizing functions from (*35*). (**E** and **F**) The distribution of the (E) canalizing strength and (F) normalized input redundancy of all observed functions with three to six essential inputs and canalizing depth 0 [that is, all functions in the blue box in (B)] is shown (blue), as well as the expected distribution for random Boolean functions (orange), derived from 1000 samples each. Horizontal lines depict the respective mean values.

To test the level of canalization in published GRN models, we stratified all 5112 update rules based on their number of essential inputs and their canalizing depth. The number of Boolean functions with a certain canalizing depth is known (*35*), and the fraction of random Boolean functions which are canalizing (i.e., those with canalizing depth ≥1) decreases exponentially as the number of inputs increases (Fig. 2A). Most identified update rules, however, had a high canalizing depth, even rules with many inputs (Fig. 2B). Four thousand eight hundred twenty-seven of the 5110 investigated update rules (94.4%) were even nested canalizing, meaning that all their variables become “eventually” canalizing (*39*). A comparison of the expected and observed proportion of canalizing and NCFs reveals the true significance of the overabundance of canalization in GRN models. These findings agree with earlier, smaller studies (*12*, *37*), which focused solely on the abundance of canalizing and NCFs but lacked the finer level of detail added by the canalizing depth.

Our findings raised an important question: Are biological networks enriched for canalizing functions solely because of the strong overabundance of NCFs, or is there broader evidence for canalization in general? To answer this, we relied on a broader mathematical definition of the biology-inspired concept of canalization, called collective canalization (*40*). Rather than focusing on single inputs that determine the output of a function regardless of the values of the remaining inputs, we studied the proportion of sets of inputs that have this canalizing ability. The recently introduced canalizing strength of a Boolean function summarizes this information in a single measure (*41*). By comparing the canalizing strength of all identified noncanalizing update rules with three to six inputs (i.e., those with canalizing depth 0) with random noncanalizing Boolean functions, we found that even those update rules, noncanalizing according to Kauffman’s stringent definition of canalization (*34*), exhibited a higher level of collective canalization than expected. Published noncanalizing update rules also exhibited more than expected input redundancy, which is an alternative measure of collective canalization (*21*).

### Redundancy

**Fig. 3.**
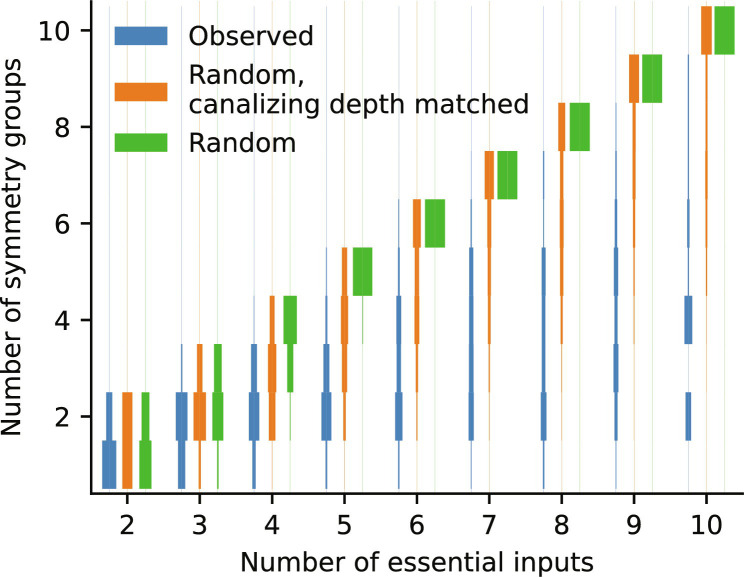
High prevalence of redundancy. The empirical distribution of the redundancy, measured by the number of symmetry groups (*y* axis), is computed for all identified update rules (blue), stratified by the number of essential inputs (*x* axis). For comparison, the expected distribution of the number of symmetry groups for random Boolean functions with 1 to 10 essential inputs is included (green), as well as the expected distribution for random Boolean functions with the same canalizing depth distribution as observed update rules (orange), as shown in Fig. 2A. Each expected distribution was generated using 1000 random functions. Figure S3 contains the explicit values of each distribution.

Genetic redundancy constitutes an important feature of gene regulation, as the presence of duplicate genes provides robustness against null mutations (*9*, *10*). We tested the level of redundancy contained in the GRN models by quantifying the number of symmetry groups for each update rule. Two regulators are in the same symmetry group if they have exactly the same effect on the targeted gene, for all possible states of all other regulators. Redundant genes perform the same function and would thus be part of the same symmetry group. We found a much higher level of redundancy in the biological networks (i.e., much fewer symmetry groups; Fig. 3 and fig. S3A) than expected by chance (fig. S3B). This comparison is skewed because canalizing functions have on average fewer symmetry groups. To exclude this confounding effect of canalization, we considered random functions whose canalizing depth was drawn from the empirical canalizing depth distribution of the published update rules (fig. S3C). Even after this correction, published models exhibited a substantially higher level of redundancy (Fig. 3).

### Feed-forward loops

**Fig. 4.**
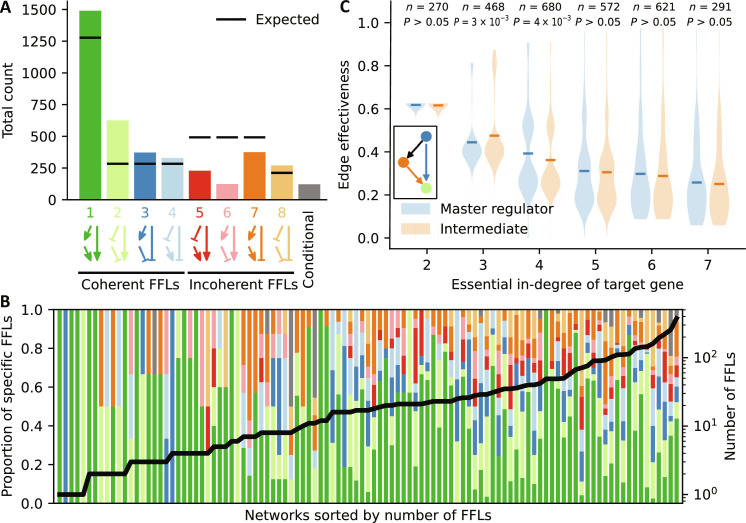
Abundance of coherent FFLs. (**A**) Total number of the different types of FFLs in the 122 GRNs (colored bars). Conditional FFLs (gray) contain at least one conditional regulation preventing the determination of their exact type. Black horizontal lines indicate the respective expected number, which is based on null model 1 (see Materials and Methods). Type 1 to 4 FFLs are coherent, while type 5 to 8 FFLs are incoherent. (**B**) Proportion [stacked bar, color-coded as in (A)] and total number (black line) of the different types of FFLs for each network. The 17 networks without any FFLs are omitted. (**C**) For each target gene in a FFL (green), the edge effectiveness of the master regulator (blue) and the intermediate regulator (orange) is compared, stratified by the essential in-degree of the target gene. Horizontal lines depict the respective mean values. *n* = number of target genes with given essential in-degree, *P* = *P* value from a two-tailed Wilcoxon signed-rank test.

Network motifs are subgraphs with a specific structure that recur throughout a network and often carry out a certain function (*3*, *4*). Several network motifs are commonly found in large, static GRN models such as the transcriptional network of *Escherichia coli* (*2*). One such motif is the FFL, which consists of three genes: one master regulator that regulates both other genes, one target gene that is jointly regulated by both others, and one intermediate gene. In a coherent FFL, the direct effect of the master regulator on the target has the same sign, either positive or negative, as the net indirect effect through the intermediate gene. Otherwise, the FFL is incoherent (Fig. 4A displays all eight FFL types). Incoherent FFLs may act as sign-sensitive accelerators of the expression of the target gene, while coherent FFLs act as sign-sensitive delays (*6*). Here, sign-sensitive means that the motif performs a function only in one direction, either when the target is up- or down-regulated.

We identified a total of 3938 FFLs in the GRN models and stratified the number of occurrences by type (Fig. 4A) and additionally by model (Fig. 4B and data S3). One hundred twenty-two FFLs (3.1%) contained conditional regulations, which means that the type of these loops changes dynamically. The expected number of activating versus inhibitory regulations contained in a FFL depends on the proportion of activating regulations in the GRN models. This proportion varies strongly from model to model (fig. S4) and decreases on average for models with higher degree. We therefore computed an expected number of each FFL type for each model, which we then summed up to obtain a total number (see Materials and Methods). Overall, the GRN models were enriched for each type of coherent FFL (Fig. 4A). This finding was consistent across kingdoms (fig. S5). All coherent FFL types, most involving two inhibitions, were almost as frequent as any incoherent FFL type, most of which contain only one inhibition. Moreover, the incoherent FFL with three inhibitory regulations (type 8) was more prevalent than two of the three incoherent FFL types with only one inhibitory regulation. It was even the only incoherent FFL, which appeared more frequently than expected.

As reported for the static GRN models of *E. coli* and *Saccharomyces cerevisiae* (*6*) and as expected by chance, the FFL with three activating edges (type 1) proved by far the most prevalent. The type 2 FFL far outnumbered the remaining FFL types, including the two other coherent ones. This is unexpected as coherent FFLs of types 2 to 4 all contain one activating and two inhibiting edges. The only potential explanation is that type 2 FFLs induce a positive effect on the target gene, while the effect is negative in type 3 and type 4 FFLs. Another interesting observation relates to type 6 FFLs. While these FFLs outnumbered all other incoherent FFLs (types 5, 7, and 8) in the static GRN models of *E. coli* and *S. cerevisiae* (*6*), we found FFL type 6 to be the least abundant. This may be due to low sample sizes in the earlier publication, or due to genuine differences in genome-wide transcriptional networks versus dynamic GRN models, which focus on a relatively small subset of genes involved in a certain biological process of interest. To explain all these observed differences, theoretical studies similar to (*8*, *42*) may be needed, which focus on the functions of the different types of FFLs in dynamic GRN models.

The target gene in a FFL is regulated by both the master regulator and the intermediate regulator. To test whether one of these two regulations is generally more important, we compared their edge effectiveness, which captures the extent to which a given input (i.e., an edge) is on average necessary to determine the value of a Boolean function (*21*); an important input has high edge effectiveness. As inputs to functions with more variables generally have lower edge effectiveness, we stratified the analysis by the essential in-degree *k* of the target gene. Albeit weakly significant but opposing differences for *k* = 3 (two-tailed Wilcoxon signed-rank test; *P* = 0.003) and *k* = 4 (*P* = 0.004), we did not find any support for the hypothesis that either the master regulator or the intermediate regulator in a FFL is generally more important.

**Fig. 5.**
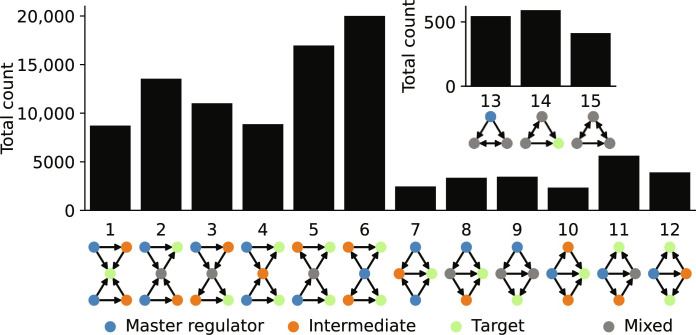
Abundance of clusters of FFLs. Total number of the different types of clusters of FFLs in the 122 GRN models. Nodes in the motif graphs are color-coded on the basis of their role in the two clustered FFLs: master regulators (blue), intermediate genes (orange), target genes (green), genes that appear in both FFLs but with a different role (gray).

We further investigated the occurrence of clusters of FFLs, that is, two FFLs that share at least one node. As with single FFLs, we can distinguish different types of FFL clusters on the basis of the distribution of activating and inhibiting edges in the motif (Fig. 5 displays all 15 types of FFL clusters). A recent analysis of a diverse set of natural and engineered networks revealed wide differences in the distribution of the different types of FFL clusters (*43*).

We identified a total of 101,832 FFL clusters in the 122 GRN models (data S4). As with the single FFL motifs, we stratified the number of occurrences by type (Fig. 5) and additionally by model (fig. S6). As expected, we found most FFL clusters to involve five genes (79,115, 77.7%), followed by four (21,168, 20.8%) and by three genes (1549, 1.5%). As in the transcriptional networks of *E. coli* and *S. cerevisiae* (*43*), type 6 was the most abundant. This type of FFL cluster features a master regulator involved in both FFLs and its abundance is likely due to the known presence of transcription factor hubs, which was also observed in this meta-analysis (Fig. 1D). Type 11 was the most abundant among all FFL clusters involving four genes. This is unexpected because transcriptional networks of *E. coli* and *S. cerevisiae* contained almost exclusively type 12 and hardly any of the other 4-gene FFL clusters (*43*). An explanation for these discrepancies likely requires novel theoretical or computational studies that relate motif structure to motif function.

### Feedback loops

FBLs constitute another important network motif. The parity of the number of inhibitory regulations determines if a FBL is positive (even number) or negative (uneven number). Each gene in a positive (negative) FBL exerts a positive (negative) effect on its own downstream expression. In general, negative FBLs buffer a perturbation and ensure homeostasis, while positive FBLs amplify perturbations and are necessary for bi- or multistationarity (*44*–*46*). We identified all FBLs involving up to six genes. For each FBL, we counted the number of activating and inhibitory regulations involved (fig. S7). Just like FFLs, some FBLs contained conditional regulations, which prevented the determination of their exact type. As expected by chance, we found more complex loops than short 2-loops or even autoregulatory loops (i.e., 1-loops). Also, FBLs with a balanced number of activating and inhibitory regulations are combinatorially more likely and were accordingly found more frequently.

To compute an expected distribution for the number of activating versus inhibitory regulations in fixed-length FBLs, we used two null models, which differ in the way that the proportion of activating regulations is computed. Null model 1 uses the same proportion for all FBLs within the same network, while null model 2 uses the fact that each FBL is contained in a strongly connected component (SCC) and derives the proportion of activating regulations only from this SCC (see Materials and Methods).

**Fig. 6.**
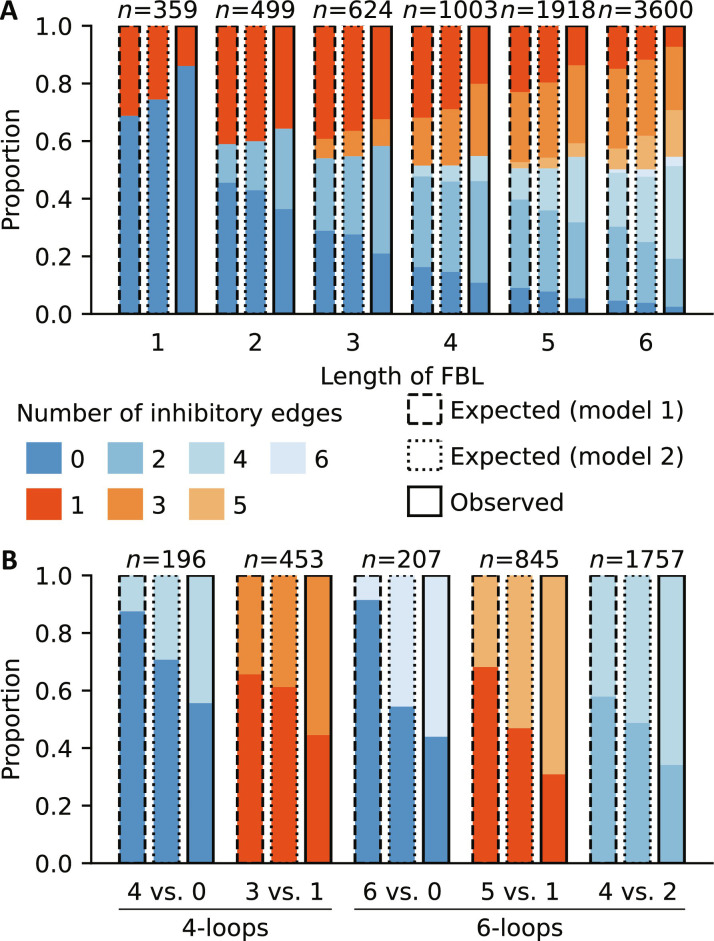
Complex feedback loops are enriched for inhibitory edges. (**A**) Stratification of all observed FBLs based on the number of involved genes (*x* axis) and the number of activating versus inhibitory edges they contain (color). Positive FBLs are blue, while negative FBLs are red. FBLs that contain conditional regulations are excluded. Each observed distribution (the rightmost of three bars with solid border) is compared to the expected distribution (left and middle bars with dashed and dotted borders), which is computed using two different null models (see Materials and Methods for details). *n* = total number of observed FBLs of a given length. (**B**) For four and six loops of the same type (positive or negative) and the same combinatorial likelihood, which depends on the number of activating versus inhibitory edges in the FBL, the observed relative abundance of FBLs with more activating versus more inhibitory edges is compared to the respective expected relative abundance, which is based on the same two null models as in (A).

For all different lengths, positive FBLs appeared slightly more frequently than expected (Fig. 6A). We also observed more self-reinforcing than self-inhibitory regulations (1-loops) than expected. On the other hand, more complex FBLs containing two or more genes were enriched for inhibitory regulations. To enable an unbiased comparison, we considered specifically complex loops of the same type (positive or negative), with the same number of genes and the same number of combinatorially expected occurrences (that is, 4-loops with 4 versus none or 3 versus 1 inhibitory regulations, or 6-loops with 6 versus none, 5 versus 1 or 4 versus 2 inhibitory regulations). All five comparisons confirmed a unexpected overabundance of negative regulations in the observed FBLs (Fig. 6B). Notably, the differences between observed and expected relative abundances were consistently smaller (but still substantial) when considering null model 2. This aligns with our finding that most SCCs that contain many FBLs have a lower proportion of activating edges than the full network (Supplementary Datasets 1 and 5). Because of insufficient numbers of FBLs in nonanimal GRN models, we were unable to assess the potential for kingdom-specific differences in the prevalence of specific types of FBLs (figs. S8 and S9).

### Criticality

Gene regulation is a highly stochastic process due to e.g. low copy numbers of expressed molecules, random transitions between chromatin states, and extrinsic environmental perturbations (*46*, *47*). While some bacteria rely on noise in gene regulation to successfully mitigate risk through bet-hedging strategies (*48*), most GRNs are incentivized to maintain a stable phenotype to ensure consistent operation of the cellular processes, despite various sources of stochasticity. At the same time, GRNs must be able to adapt to lasting changes in the environment. Because of this stability-evolvability trade-off, GRNs have been hypothesized to operate in the so-called critical dynamical regime, on the edge of order and chaos (*49*). Criticality has also been postulated for a variety of other biological networks such as neural networks or networks describing animal motion and social behavior (*50*). The dynamical robustness of a Boolean network is typically measured by the average sensitivity or more general Derrida values (*51*, *52*), which describe how a small perturbation affects the network over time. If, on average, the perturbation reduces in size after each gene has been synchronously updated once, the system operates in the ordered regime; if it amplifies on average, the system is in the chaotic regime, and if it remains, on average, of the similar size, the system exhibits criticality. Many biological systems, modeled using Boolean networks, operate in the critical regime (*12*, *53*).

**Fig. 7.**
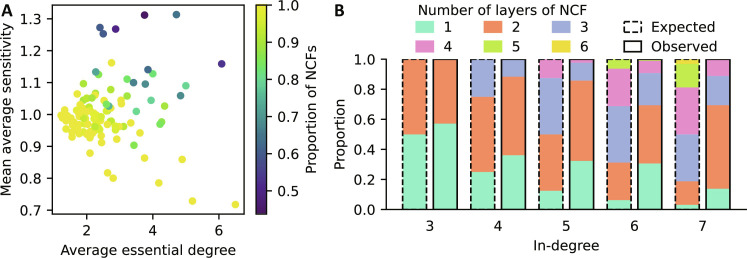
Dynamical robustness of the GRN models. (**A**) For each published model, the mean average sensitivity is plotted against the average number of essential regulators, colored by the proportion of model update rules that are NCFs. (**B**) Stratification of the observed NCFs by number of variables (*x* axis) and layer structure (colored bars). The observed relative abundance (right bars with solid borders) is compared to the respective expected relative abundance (left bars with dotted borders).

For a synchronously updated Boolean network with *N* nodes, the Derrida value for a single perturbation is simply the mean average sensitivity $s=1/N\sum_{i=1}^{N}‍S\left(f_{i}\right)$ where *S*(*f_i_*) ∈ [0, *n_i_*] is the average sensitivity of update function *f_i_* with *k_i_* inputs (*54*). For random Boolean functions in *k* (not necessarily essential) variables and with output bias *p* (which describes the probability of activation, i.e., the probability of ones in the function’s truth table), the expected average sensitivity is *2p*(1 − *p*)*k*, and thus increases linearly in *k*. On the contrary, the expected average sensitivity of NCFs is 1, irrespective of *k* (*36*). All 120 investigated models exhibited a mean average sensitivity near 1 (mean = 1.0014, SD = 0.09), which constitutes the critical threshold between order and chaos (Fig. 7A).

Across the models, mean average sensitivity was not associated with average essential degree (Pearson’s *r* = 0.03), nor with network size (Pearson’s *r* = −0.03) but depended strongly on a model’s proportion of update rules that were nested canalizing (Pearson’s *r* = −0.73; fig. S10). The eight models with the lowest mean average sensitivity (≤0.9) were all completely governed by NCFs, while the five models with the highest mean average sensitivity (≥1.17) were among the models containing the lowest proportion of NCFs (Fig. 7A).

**Fig. 8.**
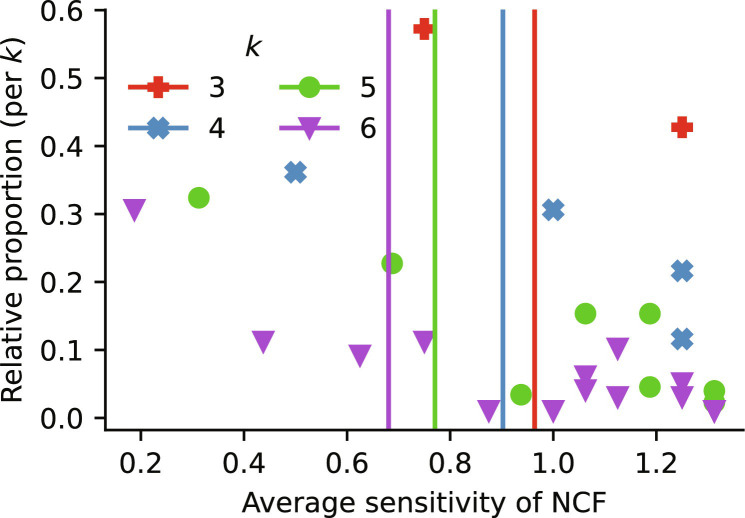
Abundance of insensitive NCFs. The relative proportion of observed NCFs in *k* = 3 to 6 variables, stratified by layer structure (exact numbers in table S2), is plotted against their average sensitivity (markers, with color differentiating *k*). For each *k*, the mean average sensitivity of all observed NCFs in *k* variables is depicted by a vertical line.

This led us to investigate the relative frequency of different NCFs in the published models. Any nonzero Boolean function has a unique standard monomial form, in which all variables are distributed into canalizing layers of importance and a noncanalizing core (*35*, *55*). NCFs are specifically those Boolean functions where the core is empty, i.e., where all variables become eventually canalizing and have a hierarchical importance order. To understand why NCFs appear frequently in GRNs, consider as an example a typical situation in gene regulation: two proteins *X* and *Y* can each independently initiate the transcription of a gene, as long as a repressor *Z* is not present to block the recruitment of RNA polymerase. The regulation of the gene in Boolean logic is best described by the NCF (*X* OR *Y*) AND NOT *Z*, which has two layers of importance, with *Z* being most important. As an example, consider again the NCF (*X* OR *Y*) AND NOT *Z*, which has two layers of importance, with *Z* being most important. NCFs with the same layer structure (i.e., with the same number of variables in each canalizing layer) have the same average sensitivity (*36*, *56*). For a given number of variables *k* ≥ 2, there exists a bijection between *p(1-p)* and the layer structure of an NCF, and there are 2^*k*−2^ NCFs with different layer structure, with each layer structure appearing equally likely by chance. Unexpectedly, we found a very nonequal occurrence among the NCFs in the published models (table S2). Partially in line with the findings of high redundancy, NCFs with fewer layers appeared more frequently (Fig. 7B). The observed NCFs also exhibited lower than expected mean average sensitivity (Fig. 8), and the higher the number of variables the lower was the observed mean average sensitivity. These findings suggest that biological networks are enriched for NCFs that induce stable dynamics as a means to counter-balance some less canalizing and more sensitive functions. While earlier studies suggested GRNs manage to operate in the critical regime due to the abundance of canalizing update rules (*12*), our results provide a more detailed understanding of this process, by pointing to NCFs with specific dynamic features as stabilizers of GRNs.

For many years, an accurate description of the critical boundary in terms of macro- and micro-level network properties has received a lot of attention. Rather than considering a binary classification problem as in (*23*, *24*), we tested how well several suggested predictors of criticality correlated with the mean average sensitivity across this largest repository of published biological networks (fig. S10). The first description of the critical boundary 2〈*k*〉〈*p*(1 − *p*)〉 = 1 (*54*), where 〈·〉 denotes the mean value across all rules within one model, only weakly correlated with the mean average sensitivity (Pearson’s r = 0.31). As described in (*12*), this is likely because it lacks to account for canalization, the essential in-degree and a negative correlation between *k* and *p*(1 − *p*) in most models. Accounting for this covariance via 〈*k*〉〈*p*(1 − *p*)〉 + Cov, as suggested in (*12*), led to a better correlation (Pearson’s *r* = 0.49). However, the covariance alone was even more correlated with the mean average sensitivity (Pearson’s r = 0.66). A predictor of the critical boundary that accounts for collective canalization by replacing *k*, the connectivity, with *K_e_*, the effective connectivity, was recently suggested: 3.94〈*K_e_*〉〈*p*(1 − *p*)〉 (*23*). This predictor correlated almost perfectly with the mean average sensitivity (Pearson’s *r* = 0.95), highlighting how well the effective connectivity captures the stabilizing effect of canalization on the dynamics of biological networks.

## DISCUSSION

Gene expression constitutes the most fundamental process in which genotype determines phenotype. A detailed understanding of the design principles that regulate this process is therefore of great importance. We used combined knowledge from numerous experts in their respective fields to perform a meta-analysis of published GRNs. Boolean networks constituted the perfect modeling framework for this kind of analysis due to their simplicity, easy comparability, and widespread use. A large literature search yielded the most extensive database of expert-curated Boolean GRN models thus far, which may be queried to generate and test various types of hypotheses.

We highlighted the usefulness of this resource by focusing on several design principles of GRNs. We confirmed that the regulatory logic is not random but highly canalized. Using a broader definition of canalization, we showed that even regulatory interactions that were not considered canalizing in previous analyses, exhibited a high level of canalization. Canalization and genetic redundancy are two correlated concepts; GRNs proved to be independently enriched for both. We further studied the presence of small network motifs and found various types of motifs that were vastly more or less abundant than expected by chance. Last, we provided strong evidence for the hypothesis that all GRNs operate dynamically close to the edge of order and chaos due to a trade-off between stability and adaptability. The abundance of nested canalizing update rules, specifically NCFs that are insensitive to perturbations, appeared to maintain critical dynamics for more densely connected GRNs.

A recent study challenges the hypothesis that most biological networks exhibit criticality (*57*). The authors argue that the abundance of external parameters (i.e., unregulated nodes) in biological network models somewhat artificially increases a network’s mean average sensitivity. When disregarding perturbations in external parameters and when considering several novel dynamical robustness metrics, many biological networks exhibit more ordered dynamics than thus far appreciated. In future work, it would be interesting to investigate how our findings, specifically the overabundance of specific classes of NCFs, affect the dynamical robustness when assessed using these novel metrics.

The described analysis suffers from several obvious limitations. First, not all biological phenomena can be accurately described in simple Boolean logic. There are a variety of published models that allow for more than two states. A similar analysis of more general models might provide more detailed insights into gene regulation but will itself suffer from the increased complexity of describing the studied concepts in the non-Boolean case. Second, there exists no feasible way to test the representativeness or completeness of our generated database of Boolean models. Even if a complete database of all published Boolean network models existed, the results would still be biased as some processes and species (e.g., model organisms) receive more attention and are modeled more frequently than others. Third, design principles of GRNs will likely differ among kingdoms of life or even among lower taxonomic levels. We therefore stratified the main analyses, wherever feasible, by kingdom. Because most of the published Boolean models and especially the large ones describe GRNs in animals, this meta-analysis lacks the statistical power to identify potential differences in design principles between kingdoms. In light of this, the identified design principles should primarily be understood as features of animal GRNs. A last limitation lies in the study design itself. Because we analyze expert-curated Boolean GRN models, it is impossible to rule out the introduction of bias by the experts who built the models. Many of the trends and properties we identified are highly significant and consistent, which means they likely reflect true biological qualities of regulatory networks. However, to know for sure, future research is needed. Because one of the main goals of synthetic biology is to generate complex networks with programmable functionality, synthetic biologists could, for example, engineer and study gene circuits that feature specific design principles suggested here. In addition, in silico experiments could clarify if and how the suggested design principles are advantageous for GRNs.

## MATERIALS AND METHODS

### Database creation

Aiming to identify all published Boolean network models of GRNs, we developed an algorithm that parses all of the more than 30 million abstracts indexed in the literature search engine Pubmed and used keywords to rank the abstracts based on how likely they were to contain a Boolean network model. To identify the keywords, we relied on the Cell Collective, a pre-existing repository of Boolean network models, which, at the time of access, contained 78 Boolean network models published in 65 distinct papers (*19*). The abstracts of these 65 papers served as a training set for the identification of keywords indicative of the presence of a Boolean network model. We considered as possible indicators (i) any word that occurred in at least two Cell Collective abstracts and was not among the most common 3000 words found in an English dictionary, (ii) all fixed combinations of two and three noncommon words like “logical modeling” or “Boolean network model”, and (iii) all co-occurrences of two or three single noncommon words in the same abstract, e.g. the co-occurrence of the words “logical”, “regulatory” and “modelling” in an abstract, not necessarily in the same fixed order. While the use of an automatic British English to American English conversion tool may have helped to limit the number of indicators, we chose to treat words that are spelled differently in British and American English as two separate words. For any possible indicator, we calculated a quality score as the ratio of the number of Cell Collective abstracts in which it occurred over the total number of Pubmed abstracts containing this indicator. This procedure resulted in 1297 publications with at least one indicator with a quality score of 5% or greater. We then manually investigated these 1297 publications to decide whether they indeed contained a GRN model. During the manual review, an additional 369 referenced publications were investigated, as they were manually deemed to be of potential interest despite lacking an indicator with quality score ≥5%, resulting in a total of 1666 reviewed publications.

### Model exclusion

To avoid the introduction of various of kinds of bias into the analysis, we used the following strict criteria for the inclusion of models.

1) We excluded models where the update rules were solely generated using an inference method or where default updates like threshold rules were consistently used. Our goal was to include only models where the update rules were built on the basis of biological expertise and knowledge gained from appropriate experiments.

2) In addition, identical models that were presented in multiple publications were only included once, and we aimed to include the earliest publication that initially presented the model. In total, 165 models passed this step and were extracted as described in the next subsection.

3) An automated quality check ensured that highly similar models were only included once in the analysis. The overlap index, also known as Szymkiewicz-Simpson coefficient, measures the overlap between two sets *A* and *B* and is defined as ∣*A* ∩ *B*∣ / min (∣*A*∣, ∣*B*∣) ∈ [0,1] (*58*). We defined two models to be highly similar if the overlap between the set of their variables (with each variable expressed as a lower case string with ‘ . ’, ‘-’, and ‘_’ removed) was ≥90%. After single-linkage hierarchical clustering of highly similar models, we manually reviewed all clusters. For each cluster, we removed all but one model from the analysis, aiming to include the final version of the model in the analysis. Most frequently, this meant inclusion of the latest published model, or the last stated model for highly similar models stemming from the same publication. This additional quality control step led to the exclusion of 39 of the 163 identified models.

4) Last, we manually investigated the overlap between all models stemming from the same publication. For one publication, we removed two additional models as a third, included model from this publication was the combination of the two excluded models (*59*). Three other publications also contained more than one model. All these models were substantially different, as they described different GRNs or pathways with low overlap between the variables (*60*–*62*).

### Model extraction and standardization

Boolean network models are presented in various formats in the literature. Using customized Python scripts, we extracted all published Boolean network models that were not excluded (see Model exclusion) and transformed them into a standardized format. In this format, each line describes the regulation of one gene; the name of the regulated gene is on the left, followed by “=”, followed by the Boolean update rule with operators AND, OR, and NOT. External parameters do not have an update rule and only occur in the update rules of the genes they regulate. For example

A=B OR C

B=A OR (C AND D)

C=NOT A

represents a model with three genes, A, B, and C, and one external parameter D.

### Meta-analysis

All analyses were performed in Python 3.10 using the libraries numpy, scipy, networkx, cana, matplotlib, and itertools. In particular, we wrote a Python script, which takes as input a Boolean model, described in standardized format, and returns, among other things, an adjacency matrix of the wiring diagram of the model, as well as completely evaluated update rules. That is, each update rule of *k* inputs is represented as a vector of length 2*^k^*, which together with the wiring diagram enables all presented analyses.

For computational reasons, we restricted most analyses to update rules with 20 or fewer inputs. The two models that each contained a single rule with more inputs (GLI1 in the hedgehog signaling pathway (*63*) is regulated by 24 inputs, while Shc in a multiscale model of ErbB receptor signal transduction (*64*) is even regulated by 27 inputs) were excluded from the network motif and criticality analyses, as the specific types of regulation (activation, inhibition, conditional) and number of essential inputs could not be determined for rules with so many inputs.

### Measures of canalization

This study includes several measures of canalization. By (*34*), a Boolean function *f*(*x*_1_, …, *x_n_*) : {0,1}*^n^* → {0,1} is canalizing if there exists a canalizing variable *x_i_*, a canalizing input *a* ∈ {0,1} and a canalized output *b* ∈ {0,1} such that

$$f\left(x_{1},\ldots ,x_{n}\right)=\left\{\begin{array}{cc} b & \text{if}\;x_{i}=a,\\ g\left(x_{1},\ldots ,x_{i-1},x_{i+1},\ldots ,x_{n}\right)\neq b & \text{otherwise} \end{array}\right.$$

If the subfunction *g* is also canalizing, then *f* is 2-canalizing, etc. More generally, *f* is *k*-canalizing, where 1 *≤ k* ≤ *n*, with respect to the permutation σ ∈ 𝒮*_n_*, inputs *a*_1_, …, *a_k_*, and outputs *b*_1_, …, *b_k_* if

$$f\left(x_{1},\ldots ,x_{n}\right)=\left\{\begin{array}{cc} b_{1} & x_{\sigma \left(1\right)}=a_{1},\\ b_{2} & x_{\sigma \left(1\right)}\neq a_{1},x_{\sigma \left(2\right)}=a_{2},\\ b_{3} & x_{\sigma \left(1\right)}\neq a_{1},x_{\sigma \left(2\right)}\neq a_{2},x_{\sigma \left(3\right)}=a_{3},\\ \vdots & \vdots \\ b_{k} & x_{\sigma \left(1\right)}\neq a_{1},\ldots ,x_{\sigma \left(k-1\right)}\neq a_{k-1},x_{\sigma \left(k\right)}=a_{k},\\ f_{C}\neq b_{k} & x_{\sigma \left(1\right)}\neq a_{1},\ldots ,x_{\sigma \left(k-1\right)}\neq a_{k-1},x_{\sigma \left(k\right)}\neq a_{k} \end{array}\right.$$

Here, *f_C_* = *f_C_*[*x*_σ(*k*+1)_, …, *x*_σ(*n*)_] is the core function, a Boolean function on *n-k* variables. When *f_C_* is not canalizing, then the integer *k* is the canalizing depth of *f* (*38*). If *k* = *n* (i.e., if all variables are become eventually canalizing), then *f* is an NCF (*65*). By (*35*), every nonzero Boolean function *f*(*x*_1_, …, *x_n_*) can be uniquely written as

$$f\left(x_{1},\ldots ,x_{n}\right)=M_{1}\left(M_{2}\left\{\cdots \left[M_{r-1}\left(M_{r}p_{C}+1\right)+1\right]\cdots \right\}+1\right)+q$$

where each $M_{i}=\prod_{j=1}^{k_{i}}‍\left(x_{i_{j}}+a_{i_{j}}\right)$ is a nonconstant extended monomial, *p_C_* is the core polynomial of *f*, and $k=\sum_{i=1}^{r}‍k_{i}$ is the canalizing depth. Each *x_i_* appears in exactly one of {*M*_1_, …, *M_r_*, *p_C_*}. The layer structure of *f* is the vector (*k*_1_, *k*_2_, …, *k_r_*) and describes the number of variables in each layer *M_i_* (*36*, *39*).

More recently, canalization has been considered as a property of the Boolean function, rather than on the variable level (*40*). In (*21*), canalization is equated to input redundancy, enabling the definition of variable/edge- and function/node-level properties, used in this study, such as the edge effectiveness and the effective connectivity. The canalizing strength constitutes an alternative approach to measure canalization on the function level (*41*). This approach generalizes Kauffman’s original definition of canalization more closely. For brevity, we refer the interested reader to these papers for details.

### Expected number of loops

The likelihood of a specific FFL or FBL type depends on the ratio of positive versus negative edges. Because of substantial variation of this ratio across models (data S1), we computed the expected distribution of specific FFL and FBL types separately for each model. For model *i*, let *p_i_* ∈ [0,1] denote the proportion of activating edges (out of all activating and inhibitory edges, excluding conditional and nonessential edges).

To compute the expected number of different FFLs in model *i*, let *n_i_* and $n_{i}^{t}$ denote the total number of FFLs and the total number of FFLs of type *t*, respectively. To create a null expectation, we assume that each edge is activating with probability *p_i_* and inhibitory with probability 1 − *p_i_*. Then

$$E\left[n_{i}^{t}|n_{i}\right]=n_{i}p_{i}^{a\left(t\right)}{\left(1-p_{i}\right)}^{3-a\left(t\right)}$$

where *a*(*t*) ∈ {0,1,2,3} denotes the number of activating edges in FFLs of type *t*. The expected number of FFLs of type *t* across all models is simply the sum of all model-specific expected numbers. This is null model 1.

Null model 1 can also be used to compute the expected number of different FBLs. Let $n_{i}^{k}$ and $n_{i}^{k,j}$ denote the total number of *k*-loops and the total number of *k*-loops containing exactly *j* inhibitory edges, respectively. Then

$$E\left[n_{i}^{k,j}|n_{i}^{k}\right]=n_{i}^{k}\left(\begin{array}{c} k\\ j \end{array}\right)p_{i}^{k-j}{\left(1-p_{i}\right)}^{j}$$

The expected number of *k*-loops containing exactly *j* inhibitory edges across all models is the sum of all model-specific expected numbers.

Null model 2 differs in the way the proportion of activating edges is computed. It uses the fact that each FBL is part of a SCC. Rather than using one overall proportion per model, null model 2 bases the expectation on the proportion within each FBL’s SCC. Let *p*_*i*,*c*_ ∈ [0,1] denote the proportion of activating edges in SCC *c* (out of all activating and inhibitory edges, excluding conditional and nonessential edges). Let $\ell_{r},r=1,\ldots ,n_{i}^{k}$ denote all *k*-loops of model *i* and let *c*(ℓ*_r_*) denote the SCC containing ℓ*_r_*. Then

$$E\left[n_{i}^{k,j},|,n_{i}^{k}\right]=\sum_{k-\text{loops}\;\ell_{r}}\left(\begin{array}{c} k\\ j \end{array}\right)p_{i,c\left(\ell_{r}\right)}^{k-j}{\left[1-p_{i,c\left(\ell_{r}\right)}\right]}^{j}$$

As before, the expected number of *k*-loops containing exactly *j* inhibitory edges across all models is the sum of all model-specific expected numbers.

### Dynamical robustness

As an indicator of the dynamical robustness of a Boolean network *F*, we computed the mean average sensitivity *s*, which describes the average size of an initial perturbation of size 1 after each gene has been synchronously updated once. That is

$$s=E\left\{d\left[F\left(x\right),F\left(y\right)\right]|d\left(x,y\right)=1\right\}$$

where *d* is the Hamming distance between two binary states. For nested canalizing networks, there exists an exact formula for *s* (*36*). For all biological networks that were not entirely governed by NCFs, we relied instead on simulations to estimate *s*. For each network, we generated 10,000 random states **x** ∈ {0,1}^*N*+*E*^ where *N* is the number of genes and *E* the number of external parameters. For each state, we selected a random gene *i* ∈ {1,2, …, *N*} to be flipped to generate **y** = **x** + **e**_**i**_ with *d*(**x**, **y**) = 1.
