# Supplementary material for: A meta-analysis of Boolean network models reveals design principles of gene regulatory networks
Source: Sci Adv. 2024 Jan 12;10(2):eadj0822. doi: 10.1126/sciadv.adj0822 (PMC10786419; doi:10.1126/sciadv.adj0822)
Supplement: Supplementary file 1 — Figs. S1 to S10 Tables S1 and S2 Legends for data S1 to S5 [file sciadv.adj0822_sm.pdf]

Science Advances
AAAS

Supplementary Materials for

A meta-analysis of Boolean network models reveals design principles of gene regulatory networks

Claus Kadelka *et al.*

Corresponding author: Claus Kadelka, ckadelka@iastate.edu

Sci. Adv. **10**, eadj0822 (2024)
DOI: 10.1126/sciadv.adj0822

The PDF file includes:

Figs. S1 to S10
Tables S1 and S2
Legends for data S1 to S5

Other Supplementary Material for this manuscript includes the following:

Data S1 to S5

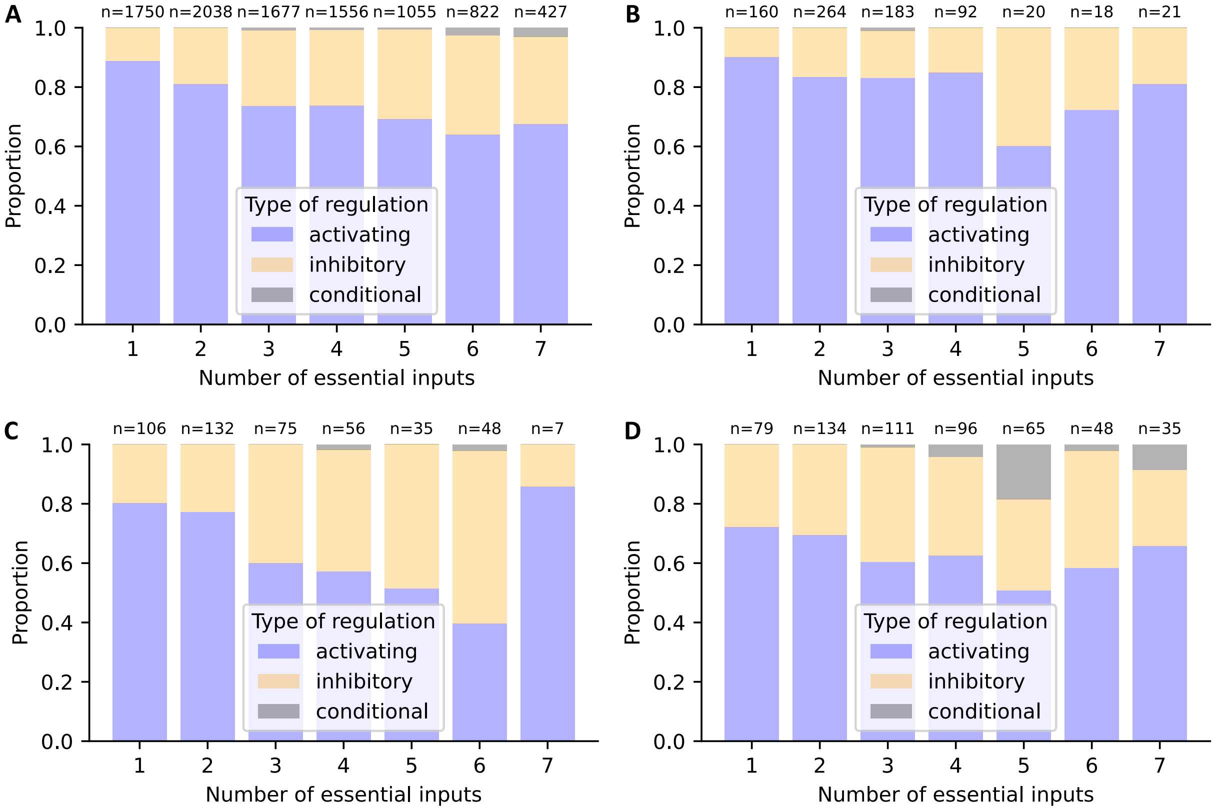

Figure S1: **Prevalence of different types of regulation per kingdom.** The prevalence of each type of regulation (activation: blue, inhibition: orange, conditional: gray) is shown. The analysis is restricted to rules with 1-7 essential inputs from Boolean GRN models of (A) animals, (B) bacteria, (C) fungi, (D) plants. The number of analyzed regulations is shown above each bar. The corresponding inter-kingdom analysis is shown in Fig. 1E.

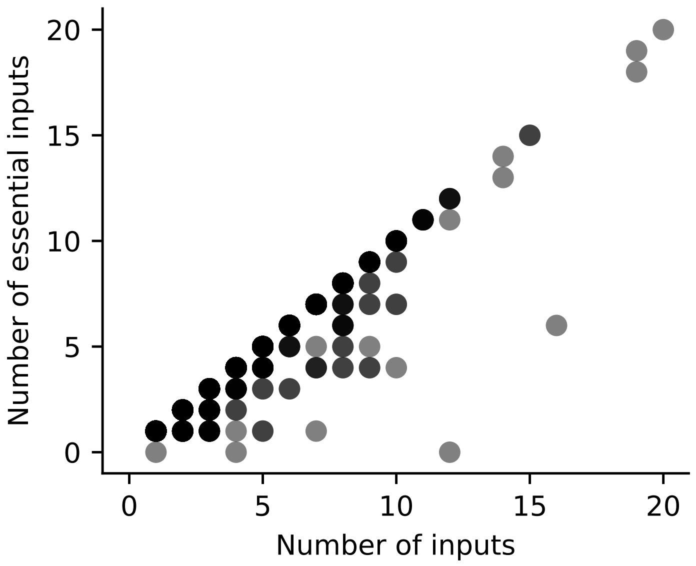

Figure S2: **Discrepancies in some published update rules**. For 5112 update rules, the number of regulators in the identified published rule (x-axis) is plotted against the number of essential inputs after simplification of the rule (y-axis).

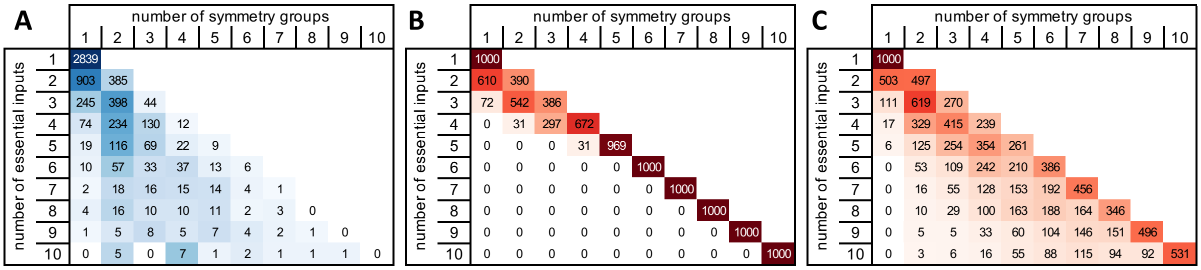

Figure S3: **Prevalence of redundancy**. (A) Stratification of all identified update rules based on the number of essential inputs (rows) and the redundancy, measured by the number of symmetry groups (columns). Update rules with more than ten essential inputs were omitted. (B-C) Expected distribution of the number of symmetry groups for random Boolean functions with 1-10 essential inputs. For each row, 1000 random, non-generated functions were generated. In (C), the distribution of the canalizing depth of the random, non-degenerated Boolean functions was matched to the one observed for the GRN models, shown in Fig. 2A.

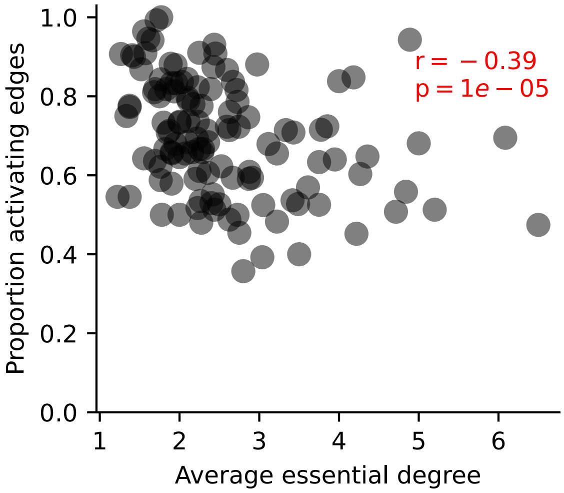

Figure S4: **Highly connected models feature a lower proportion of activating edges**. For each model, its average essential in-degree is plotted against its proportion of activating edges (out of all activating and inhibitory edges, excluding conditional and non-essential edges). The Spearman correlation coefficient and associated p-value are shown in red.

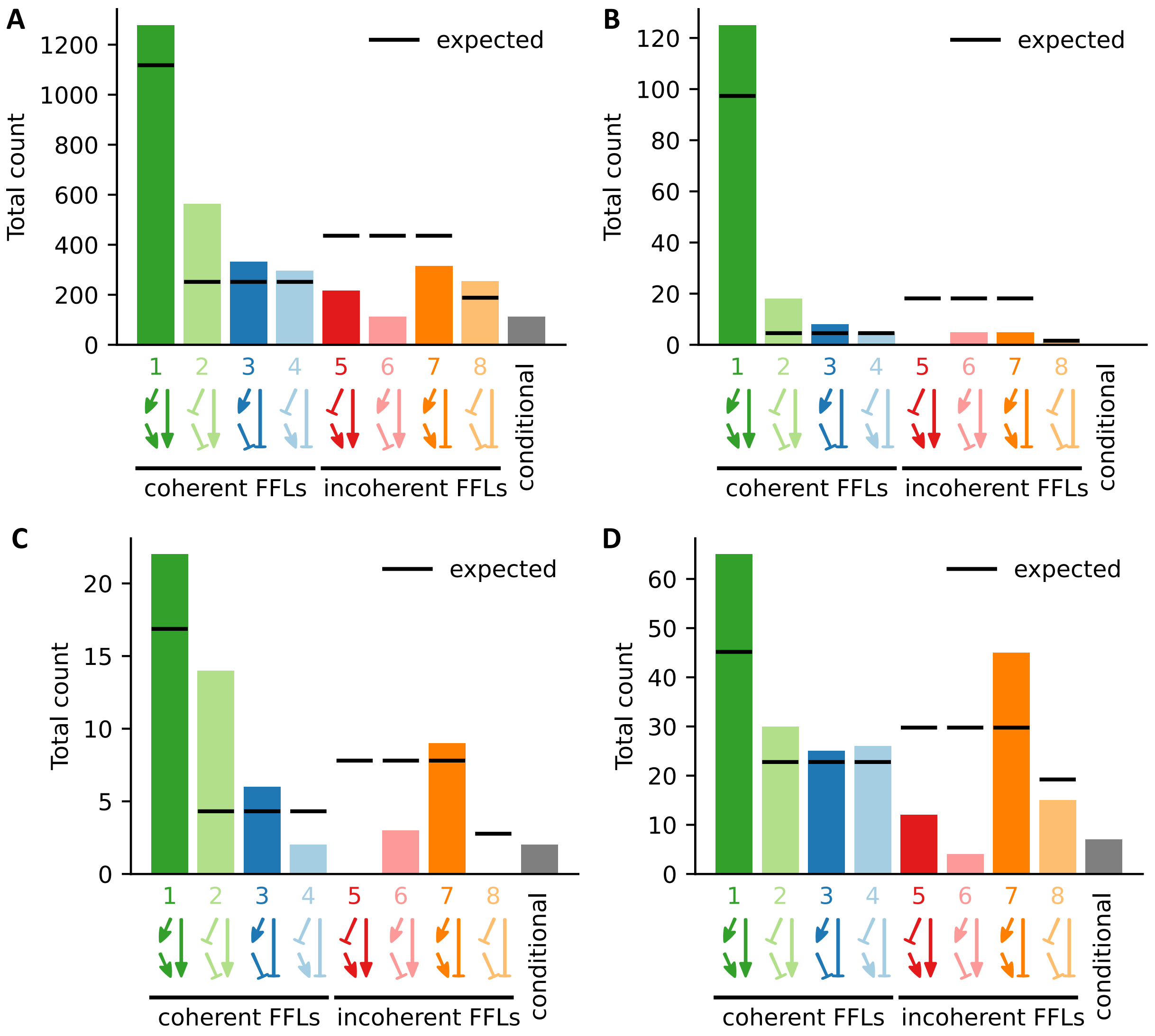

Figure S5: **Prevalence of feed-forward loops per kingdom.** The number of the different types of FFLs in GRN models of (A) animals, (B) bacteria, (C) fungi, (D) plants (colored bars) is shown. Conditional FFLs (gray) contain at least one conditional regulation preventing the determination of their exact type. Black horizontal lines indicate the respective expected number, which is based on null model 1 (see Methods). Type 1-4 FFLs are coherent, while type 5-8 FFLs are incoherent. The corresponding inter-kingdom analysis is shown in Fig. 4A.

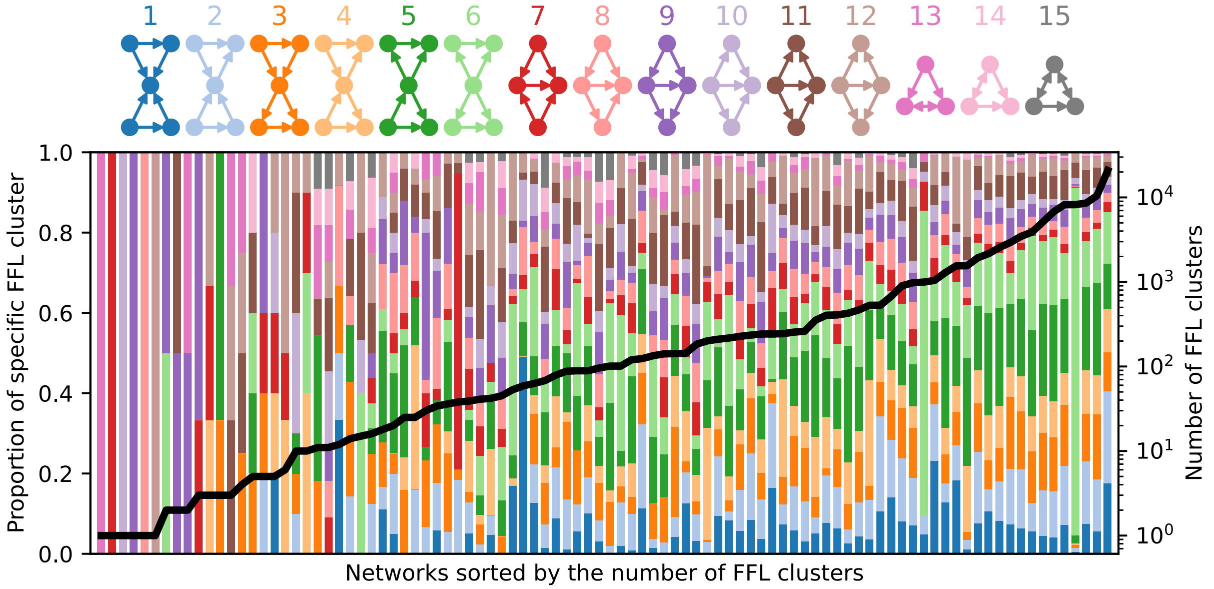

Figure S6: **Abundance of clusters of feed-forward loops per model**. Proportion (color-coded stacked bar) and total number (black line) of the different types of FFL clusters for each network. The 34 networks without any FFL clusters are omitted.

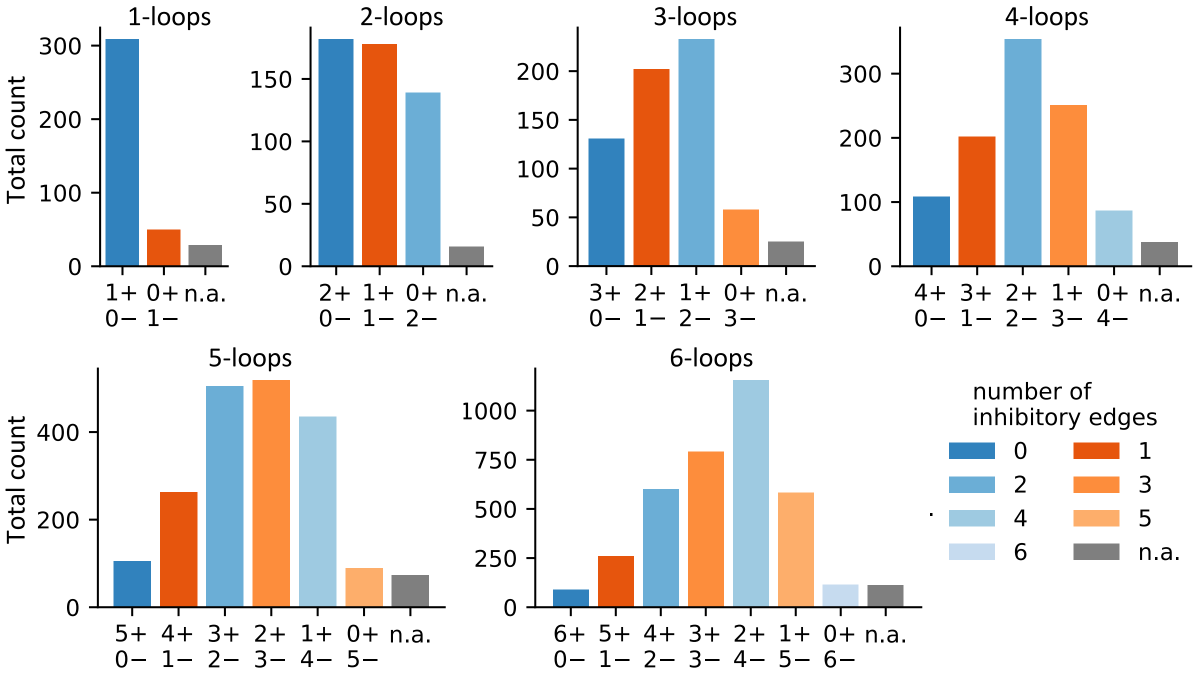

Figure S7: **Abundance of different types of feedback loops**. The total number of different types of FBLs is shown, stratified based on the number of involved genes (sub panels) and based on the number of activating (+) and inhibitory (−) regulations (x-axis). Loops, which contain conditional regulations preventing the determination of their type, are classified as n.a. Color indicates the number of negative edges in the FBL. Bars corresponding to positive FBLs (with an even number of negative interactions) are blue, while negative FBLs are red.

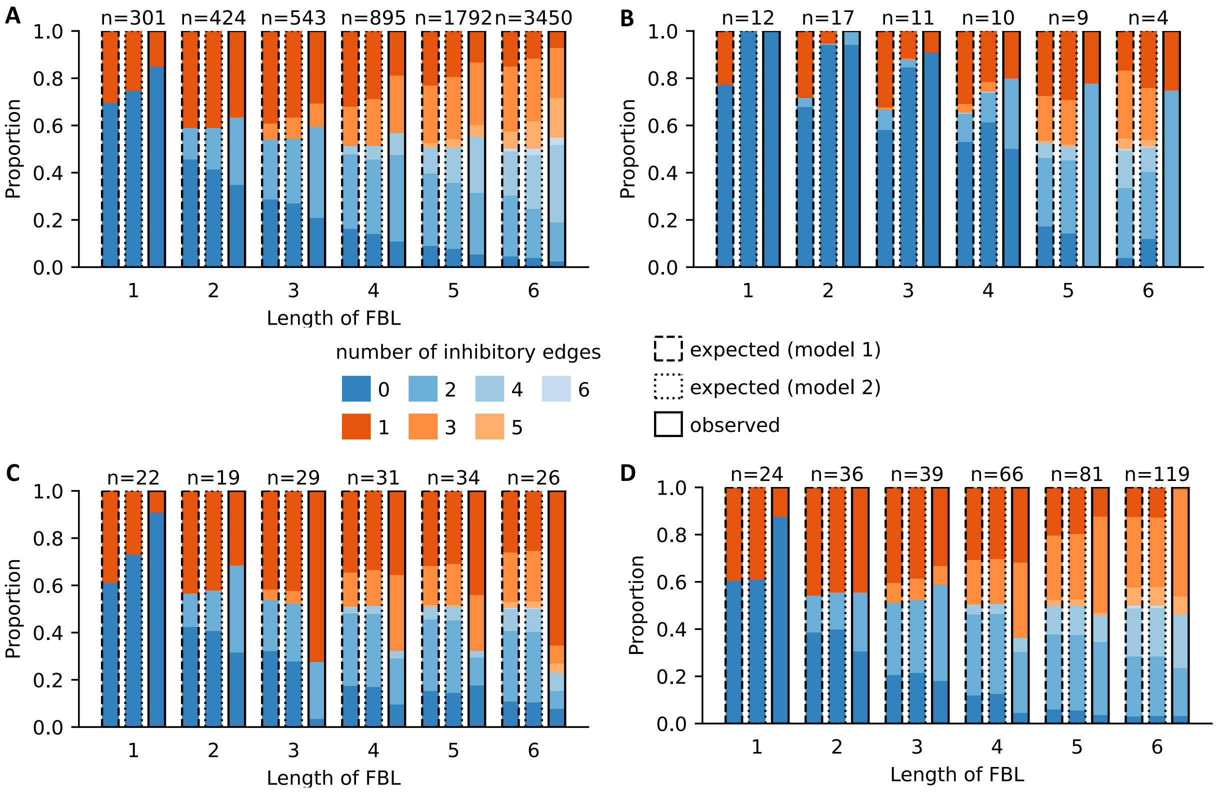

Figure S8: **Stratification of all observed feedback loops per kingdom.** All FBLs found in GRN models of (A) animals, (B) bacteria, (C) fungi, (D) plants are stratified based on the number of involved genes (x-axis) and the number of activating versus inhibitory edges they contain (color). Positive FBLs are blue, while negative FBLs are red. FBLs that contain conditional regulations are excluded. Each observed distribution (the rightmost of three bars with solid border) is compared to the expected distribution (left and middle bars with dashed and dotted borders), which is computed using two different null models (see Methods for details). n = total number of observed FBLs of a given length. The corresponding inter-kingdom analysis is shown in Fig. 6A.

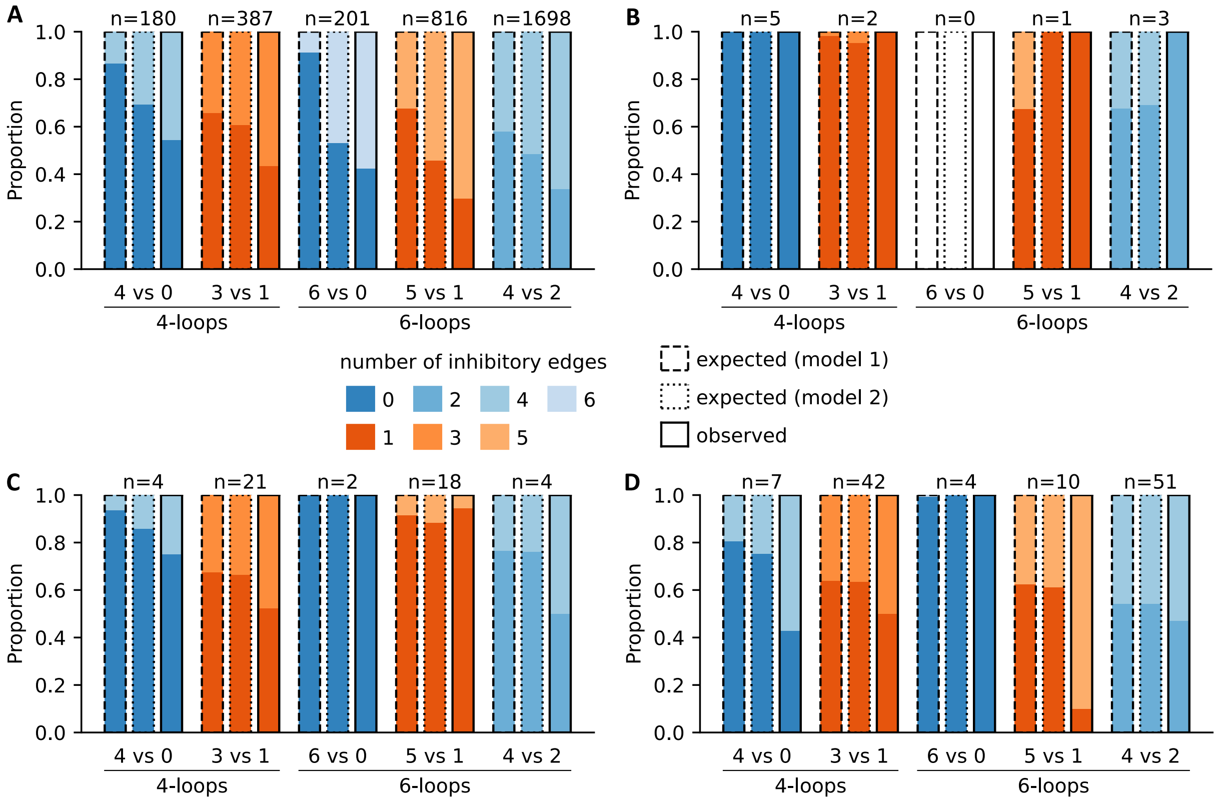

Figure S9: **Abundance of inhibitory edges in complex feedback loops per kingdom.** For FBLs of length 4 and 6 of the same type (positive or negative) and the same combinatorial likelihood, which depends on the number of activating versus inhibitory edges in the FBL, the observed relative abundance of FBLs with more activating versus more inhibitory edges is compared to the respective expected relative abundance. The expected distribution is computed using two different null models (see Methods for details). The analysis is stratified by kingdom: (A) animals, (B) bacteria, (C) fungi, (D) plants. The corresponding inter-kingdom analysis is shown in Fig. 6B.

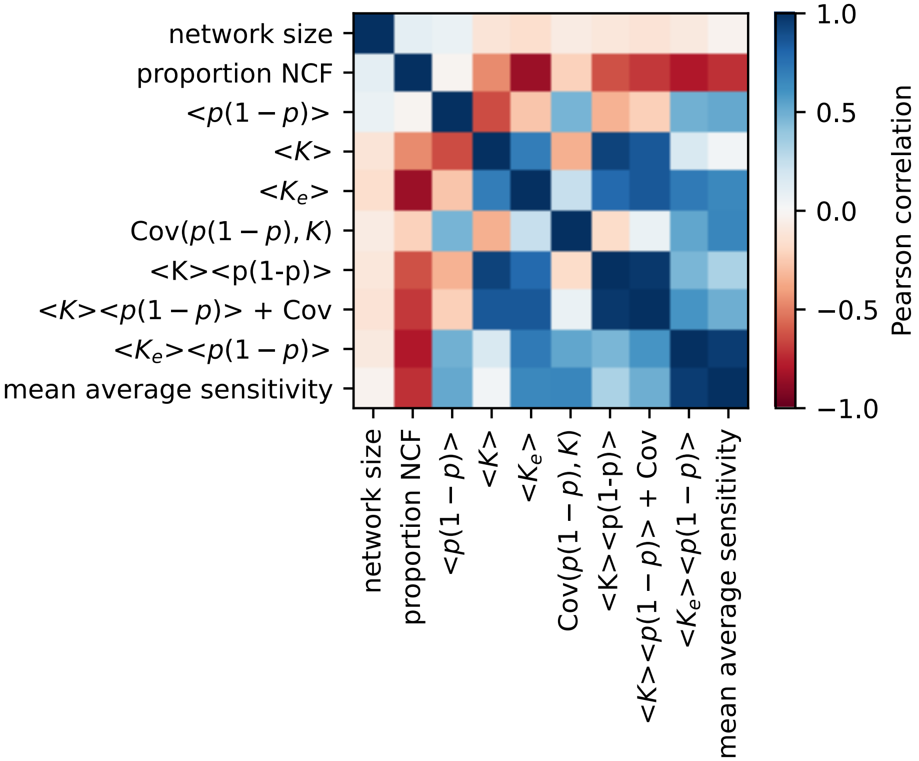

Figure S10: **Predictors of dynamical robustness**. Pairwise Pearson correlation between various properties and suggested predictors of dynamical robustness across the published GRN models. $< \cdot >$ denotes the mean, p = output bias, K = number of variables, K_e = effective connectivity, Cov = covariance of $p(1-p)$ and K.

Table S1: **High prevalence of canalization (full table)**. Stratification of all identified update rules based on the number of essential inputs (rows) and the canalizing depth (columns). The color gradient is computed separately for each row.

number of essential inputs	canalizing depth																				
	0	1	2	3	4	5	6	7	8	9	10	11	12	13	14	15	16	17	18	19	20
0	3																				
1	0	2097																			
2	0	0	1288																		
3	24	3	0	660																	
4	45	5	0	0	400																
5	33	17	3	0	0	182															
6	35	12	7	0	1	0	101														
7	16	8	6	2	1	0	0	37													
8	12	3	1	5	1	0	0	0	34												
9	6	5	5	1	2	3	0	0	0	11											
10	4	4	2	0	1	0	0	0	0	0	7										
11	3	1	0	0	1	0	0	0	0	0	0	2									
12	0	1	0	0	0	0	0	0	0	0	0	0	3								
13	0	0	1	0	0	0	0	0	0	0	0	0	0	0							
14	0	0	0	0	0	0	0	0	0	0	0	0	0	0	1						
15	0	0	0	0	1	0	0	0	0	0	0	0	0	0	0	1					
16	0	0	0	0	0	0	0	0	0	0	0	0	0	0	0	0	0				
17	0	0	0	0	0	0	0	0	0	0	0	0	0	0	0	0	0	0			
18	1	0	0	0	0	0	0	0	0	0	0	0	0	0	0	0	0	0	0		
19	0	1	0	0	0	0	0	0	0	0	0	0	0	0	0	0	0	0	0	0	
20	1	0	0	0	0	0	0	0	0	0	0	0	0	0	0	0	0	0	0	0	0

Table S2: **Observed number of NCFs with 3-6 inputs, stratified by layer structure.** The number of NCFs with a given number of inputs (3-6) and a given canalizing layer structure is shown. NCFs with the same layer structure have the same dynamical properties. All layer structures appear equally likely by chance. Green (orange) count data indicates more (fewer) than expected NCFs with a given layer structure. K_e describes the effective connectivity and p the output bias of the NCF.

k	layer structure	Hamming weight	count	count (in %)	average sensitivity	number layers	k_e	p(1-p)
**3**	3	1 or 7	372	57.2	0.75	1	1.25	0.11
	1, 2	3 or 5	278	42.8	1.25	2	1.56	0.23
**4**	4	1 or 15	137	36.1	0.50	1	1.19	0.06
	2, 2	3 or 13	116	30.6	1.00	2	1.52	0.15
	1, 3	7 or 9	82	21.6	1.25	2	1.63	0.25
	1, 1, 2	5 or 11	44	11.6	1.25	3	1.70	0.21
**5**	5	1 or 31	57	32.4	0.31	1	1.13	0.03
	3, 2	3 or 29	40	22.7	0.69	2	1.40	0.08
	2, 3	7 or 25	27	15.3	1.06	2	1.58	0.17
	1, 4	15 or 17	27	15.3	1.19	2	1.61	0.25
	1, 1, 3	9 or 23	8	4.5	1.19	3	1.73	0.20
	1, 2, 2	13 or 19	7	4.0	1.31	3	1.77	0.24
	2, 1, 2	5 or 27	6	3.4	0.94	3	1.59	0.13
	1, 1, 1, 2	11 or 21	4	2.3	1.31	4	1.80	0.23
**6**	6	1 or 63	30	30.6	0.19	1	1.08	0.02
	4, 2	3 or 61	11	11.2	0.44	2	1.28	0.04
	3, 3	7 or 57	11	11.2	0.75	2	1.45	0.10
	1, 5	31 or 33	10	10.2	1.13	2	1.58	0.25
	3, 1, 2	5 or 59	9	9.2	0.63	3	1.45	0.07
	2, 4	15 or 49	6	6.1	1.06	2	1.58	0.18
	1, 1, 2, 2	19 or 45	5	5.1	1.25	4	1.82	0.21
	2, 2, 2	13 or 51	4	4.1	1.06	3	1.68	0.16
	1, 1, 4	17 or 47	3	3.1	1.13	3	1.72	0.20
	1, 3, 2	29 or 35	3	3.1	1.25	3	1.73	0.25
	2, 1, 3	9 or 55	1	1.0	0.88	3	1.62	0.12
	1, 2, 3	25 or 39	1	1.0	1.31	3	1.82	0.24
	2, 1, 1, 2	11 or 53	1	1.0	1.00	4	1.67	0.14
	1, 2, 1, 2	27 or 37	1	1.0	1.31	4	1.82	0.24
	1, 1, 1, 3	23 or 41	1	1.0	1.31	4	1.82	0.23
	1, 1, 1, 1, 2	21 or 43	1	1.0	1.31	5	1.87	0.22

Titles of Auxiliary Supplementary Materials

Data S1 General information about the 122 models included in the meta-analysis.

Data S2 Number of occurrences of specific nodes in the 122 models included in the meta-analysis.

Data S3 Abundance of different types of feed-forward loops in the 122 models included in the meta-analysis.

Data S4 Abundance of different types of clusters of feed-forward loops in the 122 models included in the meta-analysis.

Data S5 Abundance of different types of feedback loops in the 122 models included in the meta-analysis.
